# High Frequency of Prior Severe Acute Respiratory Syndrome Coronavirus 2 Infection by Sensitive Nucleocapsid Assays

**DOI:** 10.1093/infdis/jiae174

**Published:** 2024-04-03

**Authors:** Joseph P Nkolola, Jinyan Liu, Ai-ris Y Collier, Catherine Jacob-Dolan, Yasmeen Senussi, Ella Borberg, Zoe Swank, David R Walt, Dan H Barouch

**Affiliations:** Beth Israel Deaconess Medical Center, Center for Virology and Vaccine Research, Boston, Massachusetts; Beth Israel Deaconess Medical Center, Center for Virology and Vaccine Research, Boston, Massachusetts; Beth Israel Deaconess Medical Center, Center for Virology and Vaccine Research, Boston, Massachusetts; Beth Israel Deaconess Medical Center, Center for Virology and Vaccine Research, Boston, Massachusetts; Department of Pathology, Brigham and Women's Hospital, Boston, Massachusetts; Department of Pathology, Brigham and Women's Hospital, Boston, Massachusetts; Department of Pathology, Brigham and Women's Hospital, Boston, Massachusetts; Department of Pathology, Brigham and Women's Hospital, Boston, Massachusetts; Beth Israel Deaconess Medical Center, Center for Virology and Vaccine Research, Boston, Massachusetts

**Keywords:** SARS-CoV-2, infection, nucleocapsid, serology, immunity

## Abstract

Prior infection with severe acute respiratory syndrome coronavirus 2 (SARS-CoV-2) is typically measured by nucleocapsid serology assays. In this study, we show that the Simoa serology assay and T-cell intracellular cytokine staining assay are more sensitive than the clinical Elecsys assay for detection of nucleocapsid-specific immune responses. These data suggest that the prevalence of prior SARS-CoV-2 infection in the population may be higher than currently appreciated.

The prevalence of prior severe acute respiratory syndrome coronavirus 2 (SARS-CoV-2) infection in the population remains unclear. The US Centers for Disease Control and Prevention reported in February 2022 that 75% of adults had evidence of prior SARS-CoV-2 infection [1]. Nucleocapsid serology assays are commonly utilized to detect SARS-CoV-2 infection in epidemiologic studies [1] and vaccine studies [2], because current coronavirus disease 2019 (COVID-19) vaccines only encode the spike protein and not nucleocapsid. However, it is not known whether negative nucleocapsid serology tests using the approved commercial assays accurately exclude prior SARS-CoV-2 infection, particularly given widespread population immunity and often mild clinical illness.

## MATERIALS AND METHODS

### Study Population

A specimen biorepository at Beth Israel Deaconess Medical Center (BIDMC) obtained samples from individuals who received various COVID-19 vaccines or were infected with SARS-CoV-2. The BIDMC institutional review board approved this study (2020P000361). All participants provided informed consent. This study included 22 individuals who received the bivalent messenger RNA (mRNA) boosters and included samples from September or October 2022. Participants were excluded from this analysis if they were using immunosuppressive medications.

### Roche Elecsys Assay

Elecsys Anti-SARS-CoV-2 (Roche), an approved immunoassay intended for the qualitative detection of antibodies to SARS-CoV-2 in undiluted human serum and plasma, was performed by Labcorp Inc. This assay uses a recombinant protein representing the nucleocapsid antigen for the determination of antibodies against SARS-CoV-2 and is intended for identification of individuals with an adaptive immune response to SARS-CoV-2, indicating recent or prior infection.

### MesoScale Discovery Electrochemiluminescence Assay

Electrochemiluminescence assay (ECLA) plates (MesoScale Discovery [MSD] SARS-CoV-2 immunoglobulin G [IgG], catalog number K15359 U) were designed and produced with up to 10 antigen spots in each well including SARS-CoV-2 nucleocapsid, and assays were performed as previously described [3]. The plates were blocked with 50 μL of Blocker A (1% bovine serum albumin in distilled water) solution for at least 30 minutes at room temperature shaking at 700 rpm with a digital microplate shaker. During blocking the serum was diluted to 1:5000 in Diluent 100. The calibrator curve was prepared by diluting the calibrator mixture from MesoScale Discovery (MSD) 1:9 in Diluent 100 and then preparing a 7-step, 4-fold dilution series plus a blank containing only Diluent 100. The plates were then washed 3 times with 150 μL of wash buffer (0.5% Tween in 1× phosphate-buffered saline) and blotted dry, and 50 μL of the diluted samples and calibration curve were added in duplicate to the plates and set to shake at 700 rpm at room temperature for at least 2 hours. The plates were again washed 3 times and 50 μL of SULFO-Tagged anti-Human IgG detection antibody diluted to 1× in Diluent 100 was added to each well and incubated, shaking at 700 rpm at room temperature for at least 1 hour. Plates were then washed 3 times, 150 μL of MSD GOLD Read Buffer B was added to each well, and the plates were read immediately afterward on a MESO QuickPlex SQ 120 machine. MSD titers for each sample were reported as relative light units, which were calculated using the calibrator.

### Single-Molecule Array Assay (Simoa)

A highly sensitive assay known as the single-molecule array (Simoa) multiplexed technique allows for the simultaneous measurement of IgG antibodies directed against both the SARS-CoV-2 spike and nucleocapsid, and assays were performed as previously described [4]. Human plasma samples were centrifuged at 4°C for a duration of 10 minutes at 2000*g*. The resulting supernatant underwent a 1000-fold dilution in Quanterix Homebrew Sample Diluent, prior to being employed in the assay.

### Intracellular Cytokine Staining Assay

CD4^+^ and CD8^+^ T-cell responses were quantitated by pooled peptide-stimulated intracellular cytokine staining assays. Peptide pools contained 15 amino acid peptides overlapping by 11 amino acids spanning the SARS-CoV-2 WA1/2020 membrane, nucleocapsid, envelope, or spike proteins and XBB.1.5 spike protein (21st Century Biochemicals). 10^6^ peripheral blood mononuclear cells were resuspended in 100 µL of R10 media supplemented with CD49d monoclonal antibody (1 µg/mL) and CD28 monoclonal antibody (1 µg/mL). Each sample was assessed with mock (100 µL of R10 plus 0.5% dimethyl sulfoxide; background control), peptides (2 µg/mL), and/or 10 pg/mL phorbol myristate acetate and 1 µg/mL ionomycin (Sigma-Aldrich) (100 µL; positive control) and incubated at 37°C for 1 hour. After incubation, 0.25 µL of GolgiStop and 0.25 µL of GolgiPlug in 50 µL of R10 was added to each well and incubated at 37°C for 8 hours and then held at 4°C overnight. The next day, the cells were washed twice with Dulbecco’s phosphate buffered saline (DPBS), stained with aqua live/dead dye for 10 minutes, and then stained with predetermined titers of monoclonal antibodies against CD279 (clone EH12.1, BB700), CD4 (clone L200, BV711), CD27 (clone M-T271, BUV563), CD8 (clone SK1, BUV805), and CD45RA (clone 5H9, APC H7) for 30 minutes. Cells were then washed twice with 2% fetal bovine serum/DPBS buffer and incubated for 15 minutes with 200 µL of BD CytoFix/CytoPerm fixation/permeabilization solution. Cells were washed twice with 1× Perm Wash buffer (BD Perm/Wash Buffer 10× in the CytoFix/CytoPerm fixation/permeabilization kit diluted with MilliQ water and passed through 0.22 µm filter) and stained intracellularly with monoclonal antibodies against Ki67 (clone B56, BB515), interleukin (IL) 21 (clone 3A3-N2.1, PE), CD69 (clone TP1.55.3, ECD), IL-10 (clone JES3-9D7, PE CY7), IL-13 (clone JES10-5A2, BV421), IL-4 (clone MP4-25D2, BV605), tumor necrosis factor–α (clone Mab11, BV650), IL-17 (clone N49-653, BV750), interferon gamma (IFN-γ) (clone B27; BUV395), IL-2 (clone MQ1-17H12, BUV737), IL-6 (clone MQ2-13A5, APC), and CD3 (clone SP34.2, Alexa 700) for 30 minutes. Cells were washed twice with 1× Perm Wash buffer and fixed with 250 µL of freshly prepared 1.5% formaldehyde. Fixed cells were transferred to 96-well round bottom plates and analyzed by BD FACSymphony system. Data were analyzed using FlowJo version 9.9 software.

## RESULTS

We assessed SARS-CoV-2 nucleocapsid serology in 22 participants who received 3–5 COVID-19 vaccinations, including the bivalent mRNA booster, and participated in an observational cohort at BIDMC [5] (Supplementary Tables 1 and 2). Samples were obtained in September/October 2022 and were assessed for anti-nucleocapsid antibodies by the approved Roche Elecsys immunoassay, the MSD ECLA [3], and an ultra-sensitive single-molecule array assay (Simoa) [4]. The MSD ECLA assay has a false-positive rate of approximately 3% (1 of 32 presumed uninfected early pandemic sera) [6], and the Simoa assay has a false-positive rate of approximately 1% (8 of 832 prepandemic sera) [7].

Nine of 22 (41%) participants had a clinical history of prior SARS-CoV-2 infection diagnosed by reverse-transcription polymerase chain reaction (Table 1). Ten of 22 (45%) individuals were positive by the Roche Elecsys assay, including all individuals with a clinical history of diagnosed SARS-CoV-2 infection. In contrast, 14 of 22 (64%) participants were positive by the MSD ECLA assay, and 21 of 22 (95%) individuals were positive by the Simoa assay (Table 1). Participants who were positive by the MSD ECLA and Simoa assays included all those who were positive by the Roche assay as well as most individuals who were negative by the Roche assay. In a concurrent analysis, 0 of 22 (0%) prepandemic samples were positive by the Simoa assay (Supplementary Figure 1). These data show that the MSD ECLA and Simoa assays are more sensitive than the Roche assay for detection of anti-nucleocapsid antibodies.

**Table 1.** Severe Acute Respiratory Syndrome Coronavirus 2 Infection and Nucleocapsid Serology in 22 Participants

Participant ID	Clinical Diagnosis	Roche Elecsys	MSD ECLA	Simoa
1	**+**	**+**	**+**	**+**
2	**+**	**+**	**+**	**+**
3	**+**	**+**	**+**	**+**
4	**+**	**+**	**+**	**+**
5	**+**	**+**	**+**	**+**
6	**+**	**+**	**+**	**+**
7	**+**	**+**	**+**	**+**
8	**+**	**+**	**+**	**+**
9	**+**	**+**	**+**	**+**
10	**–**	**+**	**+**	**+**
11	**–**	**–**	**+**	**+**
12	**–**	**–**	**+**	**+**
13	**–**	**–**	**+**	**+**
14	**–**	**–**	**+**	**+**
15	**–**	**–**	**–**	**+**
16	**–**	**–**	**–**	**+**
17	**–**	**–**	**–**	**+**
18	**–**	**–**	**–**	**+**
19	**–**	**–**	**–**	**+**
20	**–**	**–**	**–**	**+**
21	**–**	**–**	**–**	**+**
22	**–**	**–**	**–**	**–**

Table shows clinically diagnosed severe acute respiratory syndrome coronavirus 2 infection by reverse-transcription polymerase chain reaction and nucleocapsid serology by the approved Roche Elecsys immunoassay, the Mesoscale Discovery (MSD) electrochemiluminescence assay (ECLA), and the single-molecule array assay (Simoa).

**Figure 1.**
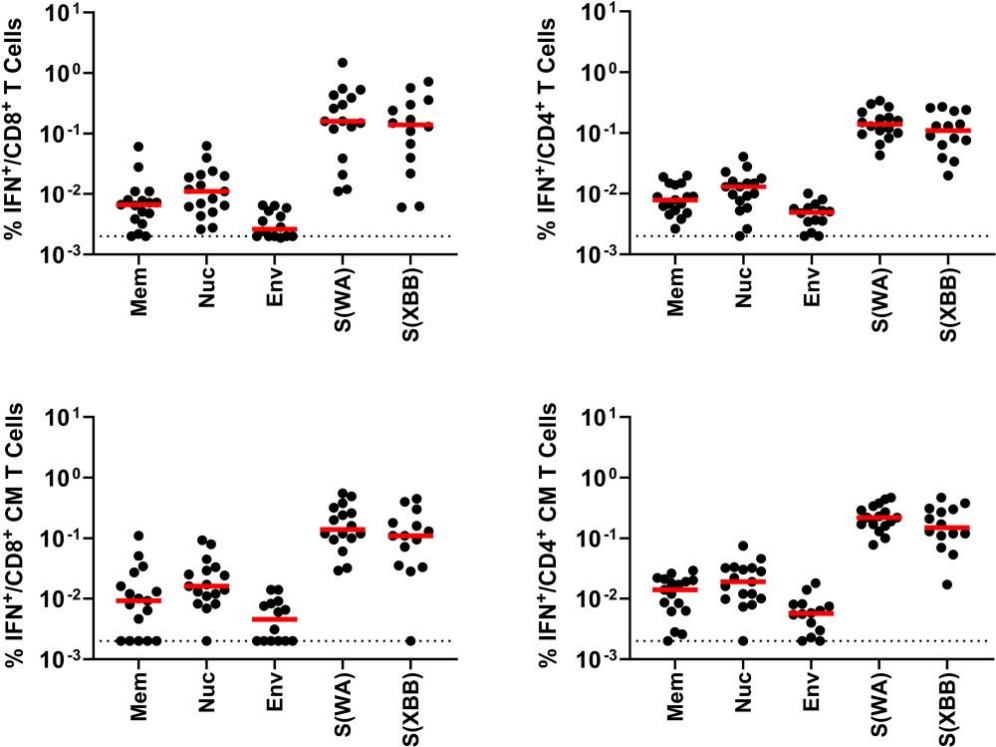
Severe acute respiratory syndrome coronavirus 2–specific T-cell responses. Antigen-specific total (top) and central memory CD27^+^CD45RA^–^ (bottom) interferon-γ CD8^+^ (left) and CD4^+^ (right) T-cell responses in the cohort described in Table 1. Cellular immune responses are shown to WA1/2020 membrane, nucleocapsid, envelope, and spike as well as XBB.1.5 spike. Dotted lines reflect limit of detection. Abbreviations: CM, central memory; Env, envelope; IFN, interferon gamma; Mem, membrane; Nuc, nucleocapsid; S, spike.

We next measured SARS-CoV-2 cellular immune responses in these individuals by intracellular cytokine staining assays [8]. Total and central memory IFN-γ CD8^+^ and CD4^+^ T-cell responses were measured to membrane, nucleocapsid, envelope, and spike proteins, which are the dominant SARS-CoV-2 T-cell targets [6, 9] (Figure 1). All participants demonstrated robust spike-specific central memory CD8^+^ and CD4^+^ T-cell responses, which reflected the combination of vaccine and natural immunity. All but 1 individual also showed nucleocapsid-specific central memory CD8^+^ and CD4^+^ T-cell responses. The participant with no detectable nucleocapsid T-cell responses was also the one who was negative by the Simoa nucleocapsid serology assay. The majority of participants also had membrane- and envelope-specific T-cell responses.

## DISCUSSION

Our data demonstrate that 21 of 22 (95%) individuals in this cohort had evidence of prior SARS-CoV-2 infection by highly sensitive nucleocapsid serology and T-cell assays. These findings suggest that the clinically approved nucleocapsid serology assays may fail to detect all individuals with prior SARS-CoV-2 infection, particularly those with mild or asymptomatic infection, although a limitation of our study is the small sample size. Nevertheless, negative nucleocapsid serology by the approved assays may not reliably exclude all cases of prior SARS-CoV-2 infection. Our study did not address the durability of nucleocapsid antibodies, which likely also wane over time.

Epidemiologic studies based on current clinical nucleocapsid serology assays may underestimate the true prevalence of prior SARS-CoV-2 infection in the population. Moreover, clinical vaccine studies may not be able to identify or exclude all individuals with prior SARS-CoV-2 infection using the approved nucleocapsid assays. Our data further suggest that some SARS-CoV-2 infections may be asymptomatic, consistent with prior studies [10]. Taken together, these data suggest that the actual prevalence of prior SARS-CoV-2 infection and the level of natural immunity in the population may be higher than currently appreciated.

## Supplementary Data

Supplementary materials are available at *The Journal of Infectious Diseases* online (http://jid.oxfordjournals.org/). Supplementary materials consist of data provided by the author that are published to benefit the reader. The posted materials are not copyedited. The contents of all supplementary data are the sole responsibility of the authors. Questions or messages regarding errors should be addressed to the author.

## Supplementary Material

jiae174_Supplementary_Data

## Notes

***Financial support*.** This work was supported by the National Institutes of Health (grant number CA260476) and the Massachusetts Consortium for Pathogen Readiness (to D. H. B.).
