# Supplementary material for: High Frequency of Prior Severe Acute Respiratory Syndrome Coronavirus 2 Infection by Sensitive Nucleocapsid Assays
Source: J Infect Dis. 2024 Apr 3;230(3):e601–4. doi: 10.1093/infdis/jiae174 (PMC11420766; doi:10.1093/infdis/jiae174)

**Table S1. Study population.**

|  | N=22 |
| --- | --- |
| **Age** (years), median (range) | 41 (25-77) |
| **Sex at birth**, Female | 14 (64) |
| **Race** |  |
| White | 19 (86) |
| Asian | 3 (14) |
| **Ethnicity** |  |
| Non-Hispanic or Latino | 22 (100) |
| **Medical condition** |  |
| Obesity (BMI $\geq$30 kg/m2) | 6 (27) |
| Hypertension | 3 (14) |
| Asthma  HIV  Pregnant | 2 (9)  2 (9)  1 (5) |
| **Prior COVID-19 vaccine history**  BNT (5 doses)  BNT (4 doses)  BNT (3 doses)/1273 (1 dose)  BNT (2 doses)/1273 (2 doses)  BNT (2 doses)/Ad26 (1 dose)/1273 (2 doses)  BNT (3 doses)/Ad26 (1 dose)/1273 (1 dose)  1273 (4 doses)  Ad26 (1 dose)/1273 (2 doses) | 3 (14)  5 (23)  5 (23)  1 (5)  2 (9)  1 (5)  4 (18)  1 (5) |
| **Days from last vaccine dose to sampling** | 22 (16-24) |
| **Known COVID-19 positive test** | 9 (40) |
| **Days from positive test to sampling*** | 191 (155-199) |

BNT=BNT162b2 or bivalent; 1273=mRNA-1273 or bivalent; Ad26=Ad26.COV2.S

Data displayed as median (range or interquartile range, IQR) and n (%); BMI, body mass index; PCR, polymerase chain reaction; pregnant designation reflects time of last vaccine dose and/or time of sampling. All individuals with known prior infection had mild disease.

*Reported for only those with known prior infection

**Table S2. Participant data.**

| **ID** | **Age** | **Sex** | **Medical history** | **Vaccine history** | **Infection history** | **Days positive test to sample** |
| --- | --- | --- | --- | --- | --- | --- |
| 1 | 25 | F |  | mRNA-1273 x 4 | After 3^rd^ dose | 170 |
| 2 | 43 | F | Obesity | mRNA-1273 x 4 | After 3^rd^ dose | 191 |
| 3 | 60 | M |  | BNT162b2 x 3, mRNA-1273 x 1 | After 4^th^ dose | 13 |
| 4 | 39 | F |  | BNT162b2 x 2, mRNA-1273 x 2 | After 3^rd^ dose | 199 |
| 5 | 56 | M | Obesity | BNT162b2 x 5 | After 3^rd^ dose | 155 |
| 6 | 27 | F |  | BNT162b2 x 3, mRNA-1273 x 1 | After 3^rd^ dose | 196 |
| 7 | 43 | M | Asthma | BNT162b2 x 3, mRNA-1273 x 1 | After 3^rd^ dose | 277 |
| 8 | 40 | M | Obesity | Ad26.COV2.S x 1, mRNA-1273 x 2 | After 3^rd^ dose | 267 |
| 9 | 50 | F | HTN | BNT162b2 x 5 | After 4^th^ dose | 121 |
| 10 | 55 | M | Asthma | mRNA-1273 x 4 |  |  |
| 11 | 34 | F | Pregnant, Gestational DM, HTN, Obesity | BNT162b2 x 4 |  |  |
| 12 | 39 | F |  | mRNA-1273 x 4 |  |  |
| 13 | 31 | F |  | BNT162b2 x 4 |  |  |
| 14 | 39 | F |  | BNT162b2 x 4 |  |  |
| 15 | 42 | F |  | BNT162b2 x 3, mRNA-1273 x 1 |  |  |
| 16 | 37 | F |  | BNT162b2 x 2, Ad26.COV2.S x 1, mRNA-1273 x 2 |  |  |
| 17 | 68 | M |  | BNT162b2 x 3, Ad26.COV2.S x 1, mRNA-1273 x 1 |  |  |
| 18 | 77 | F | Obesity | BNT162b2 x 4 |  |  |
| 19 | 25 | M |  | BNT162b2 x 4 |  |  |
| 20 | 45 | F |  | Ad26.COV2.S x 1,  BNT162b2 x 2,  mRNA-1273 x 2 |  |  |
| 21 | 62 | M | Obesity, HTN | BNT162b2 x 5 |  |  |
| 22 | 32 | F |  | BNT162b2 x 3,  mRNA-1273 x 1 |  |  |

HTN, hypertension; DM, diabetes mellitus.

**Figure S1. Simoa nucleocapsid serology raw data.** Data from test samples and 22 pre-pandemic samples are shown. Dotted line reflects limit of detection.


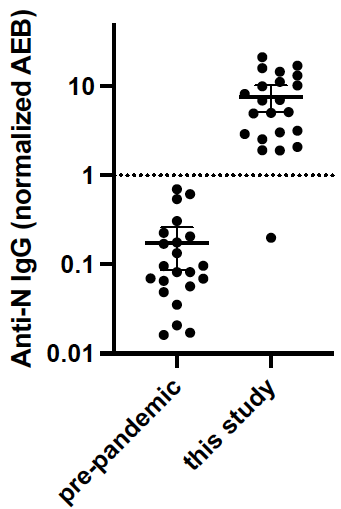

Supplement: jiae174_Supplementary_Data [file jiae174_supplementary_data.docx]
